# Taxonomic and functional shifts of gut microbiome in immunoglobulin A vasculitis children and their mothers

**DOI:** 10.3389/fped.2024.1356529

**Published:** 2024-02-12

**Authors:** Yijia Liang, Changying Zhao, Lanlan Zhao, Dashuang Sheng, Bin Chen, Guoping Zhao, Qinghua Wang, Lei Zhang

**Affiliations:** ^1^ Microbiome-X, School of Public Health, Cheeloo College of Medicine, Shandong University, Jinan, China; ^2^ State Key Laboratory of Microbial Technology, Shandong University, Qingdao, China; ^3^ CAS Key Laboratory of Computational Biology, Bio-Med Big Data Center, Shanghai Institute of Nutrition and Health, University of Chinese Academy of Sciences, Chinese Academy of Sciences, Shanghai, China; ^4^ School of Biological Science and Technology, University of Jinan, Jinan, China

**Keywords:** gut microbiota, immunoglobulin A vasculitis, microbial pathway, mother-child pairs, bacterial biomarker

## Abstract

**Objectives:**

To examine the gut microbiota characteristics in children with immunoglobulin A vasculitis and their interrelationships with the host, while evaluate the vertical inheritance of microbiota in the development and progression of IgA vasculitis.

**Methods:**

This study investigated the gut microbiome of 127 IgA vasculitis mother-child pairs and 62 matched healthy mother-child pairs, and compared the gut microbial composition of different groups. The pathway enrichment analysis evaluated potential gut microbiome-mediated pathways involved in the pathophysiology of IgA vasculitis. The Spearman correlation analysis illustrated the relationships between clinical variables and bacterial biomarkers.

**Results:**

This study identified distinct intestinal microbiome in IgA vasculitis children compared to healthy children, and further pointed out the association in gut microbiota between IgA vasculitis children's and their mother's. The relative abundance of *Megamonas* and *Lactobacillus* in IgAV children was positively correlated with that in their mothers. The pathway enrichment analysis found microbial biosynthesis of vitamins and essential amino acids was upregulated in children with IgA vasculitis. Correlation analysis showed bacterial biomarkers were correlated with indicators of blood coagulation.

**Conclusion:**

Children with IgA vasculitis have unique bacterial biomarkers and may affect coagulation function, and their gut microbiome was closely associated with that of their mothers. The observed association in gut microbiota between IgA vasculitis children and their mothers suggested a potential intergenerational influence of the maternal microbiota on the development or progression of IgA vasculitis in children.

## 1 Introduction

Immunoglobulin A vasculitis (IgAV) is the most common type of primary vasculitis in children (1). In clinical terms, IgA vasculitis is characterized by symptoms such as abdominal pain, deposition of IgA in biopsy samples, arthritis or joint pain, and the presence of hematuria or proteinuria (2). IgA vasculitis typically follows a self-limiting course, with symptoms resolving within a few weeks to months. However, one-third of pediatric patients have a recurrence of the disease with the highest prevalence of recurrence observed in children between the ages 4 and 6 (3). Close monitoring is essential, particularly in cases with renal involvement, to prevent long-term complications. The pathogenesis of IgAV remains unclear (4). Many studies have confirmed that the prevalence of IgAV aligns with the epidemiological features of certain pathogenic microorganisms (5, 6).

The gut microbiome is widely believed to have a close relationship with human health, and is thought to have significant impacts on the autoimmune disease (7, 8). Gut-associated IgAV symptoms, such as abdominal pain, are discussed to be dependent on changes in the composition and metabolic functions of the gut microbiota (9). There is, in fact, increasing evidence suggesting that bacterial dysbiosis may exert a significant influence on the development of IgAV (4, 10, 11). However, the specific mechanisms by which the gut microbiota affects IgAV development and progression are still obscure (4). In addition, maternal microbiome is also a non-genetic factor associated with disease susceptibility in offspring (12, 13). Many studies have confirmed that maternal regulation of offspring's health through gut microbial metabolites (14, 15). The changes in maternal microbiota could be transmitted to the offspring at various stages such as prenatal or postnatal, potentially resulting in an altered microbiota in the offspring. These microbial alterations in the offspring have been associated with an elevated susceptibility to non-communicable diseases (NCDs), including allergy-related issues (16).

In our previous study, dysbiosis of the gut microbiota was observed in children with IgAV (17). However, there has been no study investigated the microbial composition of IgAV mother-child pairs or evaluated the disease correlations of mother-child microbial dysbiosis. Hence, this study compares the gut microbial composition of IgAV children and their mothers, while also evaluating any potential gut microbiome-mediated pathways involved in the pathophysiology of IgAV. To expand our understanding, we explored the microbiomes of study participants through two sequencing approaches. Firstly, we directly assessed the gut microbiota of 127 IgAV mother-child pairs and 62 matched healthy mother-child pairs using 16S ribosomal RNA (16S rRNA) gene sequencing on their fecal samples, and compared the overall microbiome composition as well as the relative abundance of specific bacterial taxa among different groups. Furthermore, we investigated whether the gut microbiota's metabolic pathways were altered in IgAV, potentially explaining its mechanisms of action.

## 2 Materials and methods

### 2.1 Study design and population

In this study, patients were recruited from the Children's Hospital Affiliated to Shandong University between July 2016 and November 2019. IgAV children were enrolled per the following criteria: (a) symptoms and physical indicators that fulfill the diagnostic criteria (18) for IgA vasculitis in a clinical setting; (b) systemic treatment has not yet commenced in the acute stage of the disease; (c) no use of antibiotics or microecological preparations in the last three months. We collected fecal samples from 127 children who were diagnosed with IgA vasculitis and met the enrollment criteria. All samples were collected at the time of initial hospitalization prior to systemic treatment. The samples were gathered in sterile centrifuge tubes and subsequently stored at a temperature of −80°C for preservation. Controls were recruited among age-matched healthy children, and a thorough physical examination was conducted to confirm the absence of any genetic history or signs of inflammation. All the mothers were not pregnant, without IgAV, tested negative for HIV, had no clinically evident inflammatory conditions, and had not used any antibiotics within a five-week period preceding the initiation of the study. After collecting stool specimens, blood tests and urinalysis were conducted on children with IgAV to reflect their immunity, blood coagulation, and nephritis status.

The original clinical studies were approved by the Ethics committee of the Children's Hospital Affiliated to Shandong University. All the written informed consent were signed by parents before the study.

### 2.2 Sample collection and DNA extraction

Sterilized 2 ml tubes containing pure ethanol were used to collect stool samples from the enrolled subjects. The samples were then immediately frozen at −20°C and within three days, they were transferred to a −80°C freezer for storage. Fecal microbial genomic DNA extraction was performed following the same method as previously described (17).

### 2.3 Bacterial 16S rRNA gene amplicon sequencing

To investigate the microbial community in the samples, amplification of the bacterial 16S rRNA gene's V1-V2 hyper-variable region was conducted. The amplification employed two universal bacterial 16S rRNA gene amplicon PCR primers(PAGE purified): forward primer-27F (5’-*AGAGTTTGATCMTGGCTCAG*-3’) and reverse primer-355R (5’-*GCTGCCTCCCGTAGGAGT*-3’). The amplicons were purified using the QIAquick PCR Purification Kit (Qiagen) following the PCR purification procedure. Subsequently, all amplicons were quantified and combined to standardize concentrations for sequencing on the HiSeq 2,500 platform (Illumina).

### 2.4 16S rRNA gene sequences processing and analysis

The raw sequencing data underwent processing and analysis utilizing the Quantitative Insights into Microbial Ecology 2 (QIIME2, version2020.2). In summary, paired-end reads were matched to their respective samples based on barcodes, after which both the barcodes and primer sequences were removed. Next, the q2-dada2 (19) plugin in QIIME2 was utilized for quality control, detection and removal of chimeras, as well as the generation of amplicon sequence variants (ASVs) along with their representative sequences. We used the SILVA database (version 138) (20) classifier to annotate the ASVs based on a 99% similarity threshold. Samples that had fewer than 10 ASV features and fewer than 10,000 reads, as well as representative sequences with a frequency of less than 10, were excluded from further analysis.

We utilized the get_alphaindex function from the MicrobiotaProcess R package (version 1.2.0) to calculate alpha diversity. For beta diversity analysis, the Bray-Curtis distance was computed using the vegdist function in the vegan R package (version 2.5-7) following normalization through Hellinger transformation. To compare differences between the two groups, the Wilcoxon rank sum test was used for alpha diversity, while the permutational multivariate analysis of variance (PERMANOVA) was applied for beta diversity. Additionally, the diff_analysis function in the MicrobiotaProcess R package was used to conduct Linear Discriminant Analysis (LDA) Effect Size (LEfSe) analysis (LDA >2), in order to identify the differential microbiota between the two groups. The Venn diagram based on the ASVs and the heatmap of the Spearman correlation were also generated using the R software.

### 2.5 Metagenomics sequencing and data analysis

The DNA libraries for the metagenomics of 55 fecal samples from children were constructed using DNA extraction kits and were then sequenced using the Illumina Nova Seq 6,000 system. The resulting reads were processed using the bioBakery 3 tools (21), which included preprocessing with Kneaddata, taxonomic analysis with MetaPhlAn3, and functional analysis with HUMAnN3. The DIAMOND aligner was used to map reads to the UniRef90 database by HUMANn3 to identify the UniRef protein families. We used the humann_renorm_table script to normalize the reads per kilobase output from HUMAN3 to relative abundance data, and the data was used as input for STAMP.

### 2.6 Phylogenetic analysis

The complete sequences of all ASVs in the four shared genera obtained in this study were selected for phylogenetic analysis. The Neighbor-Joining method of MEGA_11 was employed to process the analysis with 1,000 bootstrap replicates.

## 3 Results

### 3.1 Clinical data of the study population

127 IgAV children (IgAV-C, *n* = 127) and their mothers (IgAV-M, *n* = 127), together with 62 healthy children (H-C, *n* = 62) and their mothers (H-M, *n* = 62) were enrolled in this study. We carried out 16S rRNA gene sequencing from their fecal samples. The average ages of IgAV children (IgAV-C) and healthy controls (H-C) were 6.1 ± 2.5 years and 5.8 ± 2.7 years, respectively. The ratio of male to female is 1:0.477 and 1:0.879. There was no significant difference in age (*p* = 0.452) and gender (*p* = 0.076) among the two groups. There was also no significant difference in age (32.6 ± 4.8 years vs. 33.5 ± 4.5 years, *P* = 0.208) between IgAV-M and H-M groups. Meanwhile, We randomly selected 28 individuals from IgAV children (disease group) and 27 individuals from healthy children (healthy group), whose samples were simultaneously performed on metagenomic sequencing. These two groups were matched for age (7.4 ± 2.1 years vs. 7.0 ± 3.2 years, *P* = 0.585) and gender (1:0.647 vs. 1:0.5, *P* = 0.858).

Common clinical symptoms of IgAV in children included tangible rashes on the limbs (76.47%), gastrointestinal symptoms (47.06%), arthritis (43.14%), and renal damage (18.11%). All IgAV children had not used probiotics, antibiotics, hormonal or immunosuppressive drugs for at least 3 months. Other laboratory test indicators for each IgAV child are shown in the Supplementary Data 1.

### 3.2 A landscape of IgAV-C and IgAV-M's gut microbiome revealed by 16S rRNA gene amplicon sequencing

**Figure 1:**
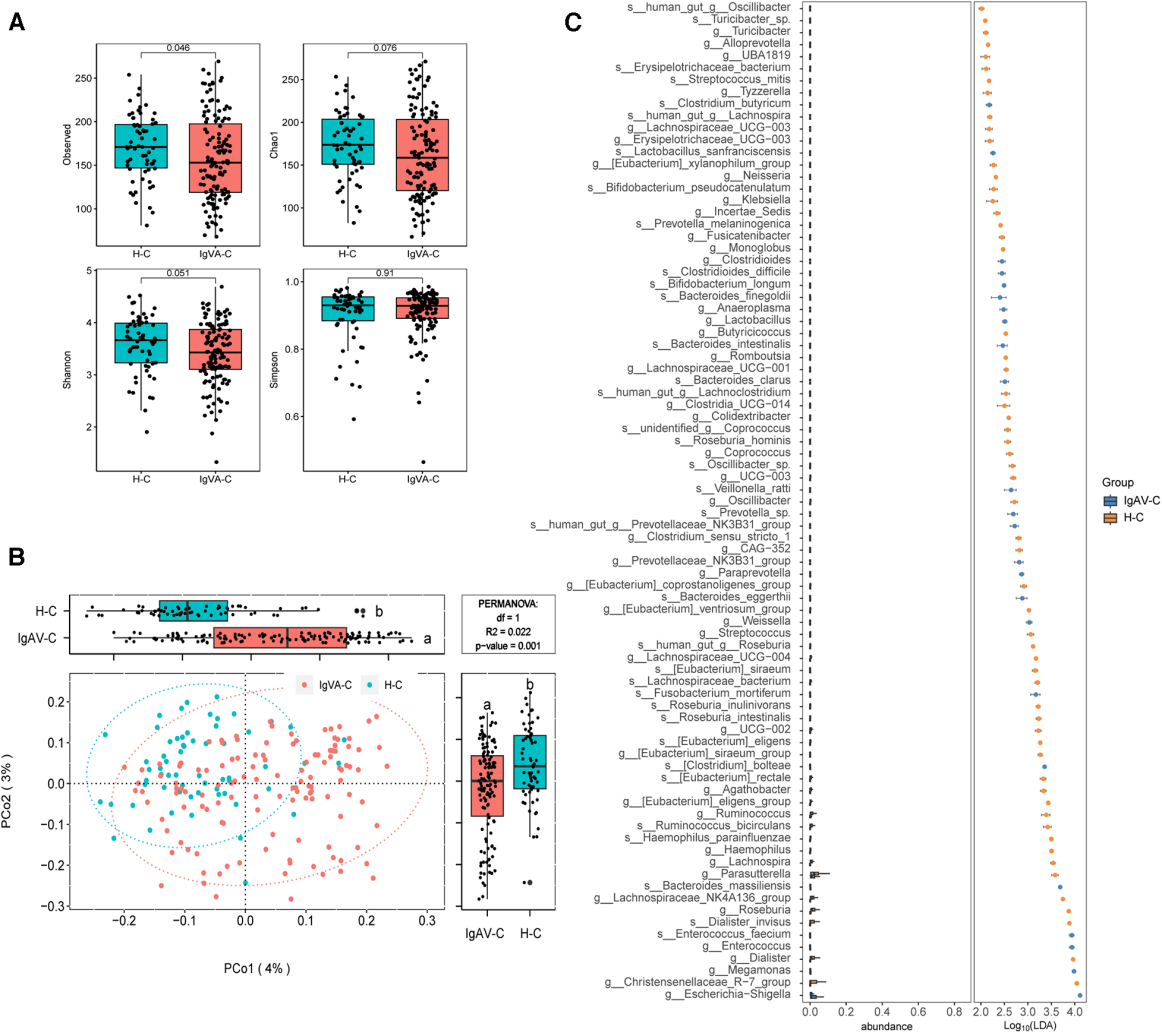
Distinct gut microbiomes were observed in IgAV children (IgAV-C) compared to healthy controls (H-C). (**A**) Four indexes of α-diversity representing the abundance and evenness of the gut microbiota, in the intestinal microbiome compared between IgAV-C and H-C groups. H-C group was shown in blue and IgAV-C group was shown in red. (**B**) PCoA and boxplot are shown along the first two principal coordinates of Bray-Curtis distances for IgAV-C and H-C. Ellipses represent the 95% confidence interval around the group centroid. The *P* value was calculated by PERMANOVA. Different letters in the boxplot indicate significant differences between the two groups. (**C**) Significantly different abundant taxa with LDA score (log10) >2.0 and *P* < 0.05 at the species or genera level, between IgAV-C and H-C groups.

To assess the gut microbiome of children with IgA vasculitis (IgAV), we investigated the alpha diversity by comparing IgAV-C and H-C groups. The result revealed a marked reduction in microbial richness, indicating a significant decrease in diversity within the gut microbiota of children with IgAV (Observed index, Figure 1A). The beta diversity analysis based on the Bray-Curtis distance revealed distinguishable gut microbiome compositions between IgAV-C and H-C groups (*P* = 0.001, Figure 1B). In order to gain deeper insights into the gut microbial community characteristics of IgAV-C, a comparative analysis of the relative taxon abundance was performed between IgAV-C and H-C groups. LEfSe analysis found 120 biomarkers that effectively differentiated the gut microbial communities of IgAV children from those of healthy children, with a Linear Discriminant Analysis (LDA) score exceeding 2. Among these taxa, 34 were identified as enriched in children with IgAV, while 86 taxa were found to be enriched in healthy children (LDA >2; Supplementary Data 2). At the genus level, 9 genera, such as *Enterococcus*, *Lactobacillus*, *Megamonas* and *Clostridioides*, were found to be more abundant in children with IgAV (Figure 1C). Taken together, our results indicated the alterations in gut microbiome had a connection with IgAV.

**Figure 2:**
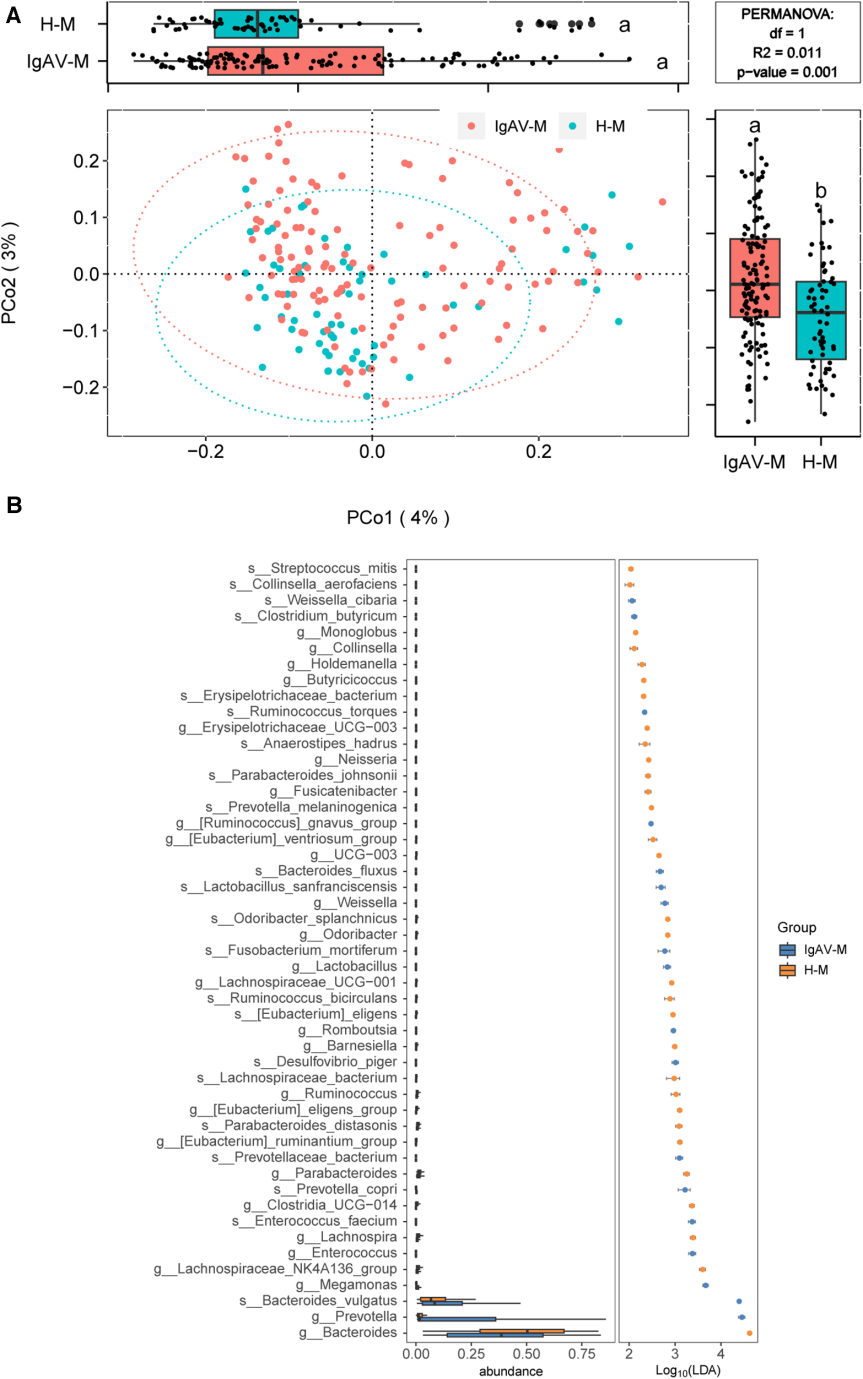
Distinct gut microbiomes were observed in IgAV-M compared to H-M. (**A**) PCoA and boxplot are shown along the first two principal coordinates of Bray-Curtis distances for IgAV-M and H-M. Ellipses represent the 95% confidence interval around the group centroid. The *P* value was calculated by PERMANOVA. Different letters in the boxplot indicate significant differences between the two groups. The results showed significant differences in the bacterial community structure between the two groups (**B**) Significantly different abundant taxa with LDA score (log10) >2.0 and *P* < 0.05 at the species or genera level, between IgAV-M and H-M groups.

We further investigated the distinction of microbiota compositions among IgAV-M and H-M. The analysis of beta diversity indicated a significant difference between the microbiome of IgAV-M and H-M (*P* = 0.001, Figure 2A). LEfSe analysis identified 49 species or genera that exhibited significant differences between IgAV-M and H-M (Figure 2B).

### 3.3 The gut microbiomes of IgAV children and their mothers exhibit a close association

**Figure 3:**
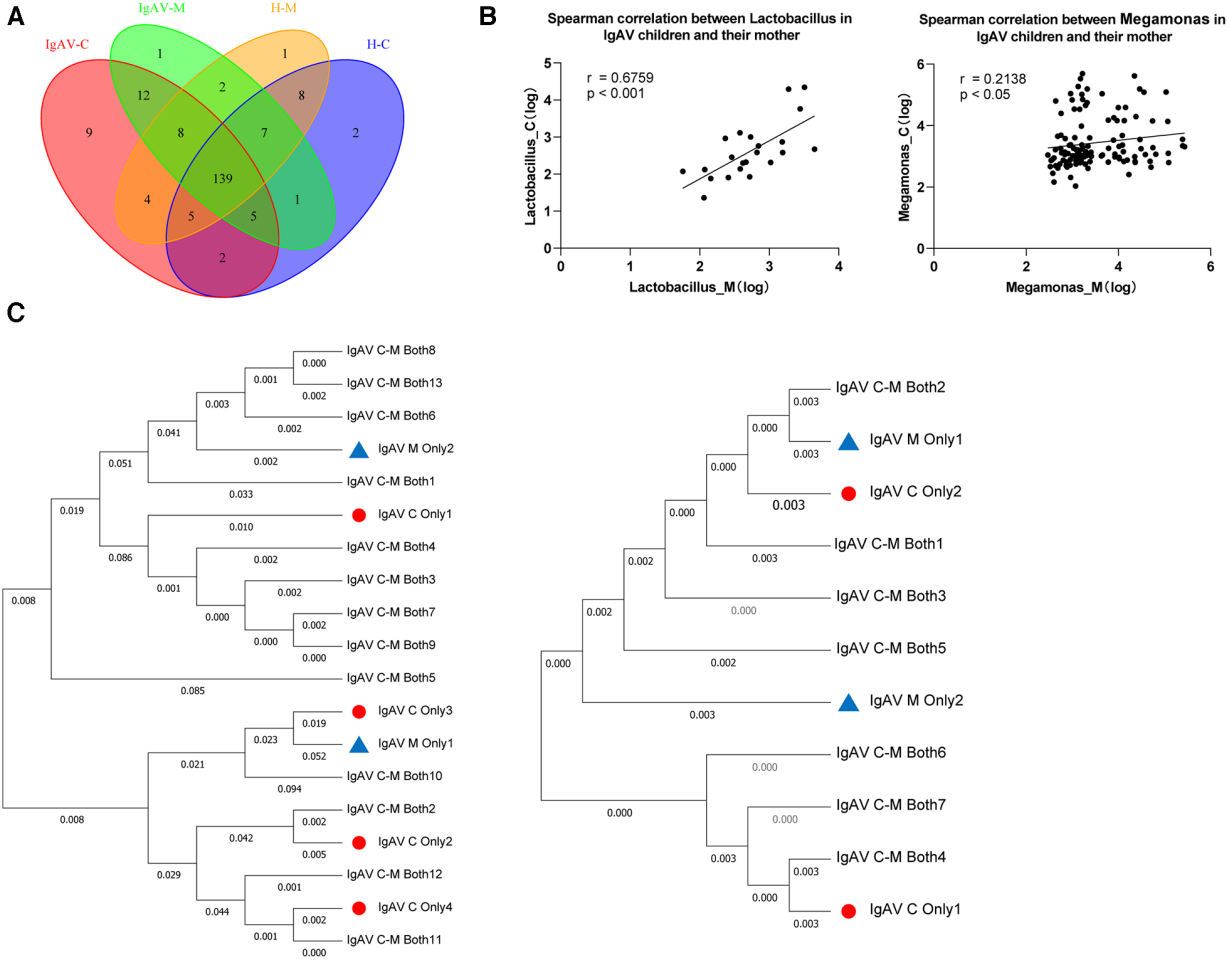
Association between gut microbiome in IgAV children and their mothers. (**A**) Venn diagram displaying the degree of overlap of bacterial ASVs among IgAV-C, IgAV -M, H-C and H-M. Venn diagrams were constructed to evaluate the number and identity of the shared ASVs among groups. (**B**) Spearman correlation between abundance of specific taxa in IgAV-C and IgAV-M. (**C**) Mother-child phylogenies for *Lactobacillus* and *Megamonas*. ASVs, only in IgAV-C were represented by triangles and only in IgAV-M by circles. ASVs both in IgAV-M and IgAV-C were labeled with the specific ID in the trees. *Lactobacillus* at left and *Megamonas* at right. (**A-C**), demonstrating that the similarity of the gut microbiome in the IgAV-M + C group.

Subsequently, we employed Venn diagrams to display the similarities and distinctions among the four groups. There is a most similarity gut microbiome composition between children and the their mothers whether the disease or control, as well as each groups contained their own particular microbiome (Figure 3A). Accordingly, we found 12 genera such as *Abiotrophia* and *Clostridioides*, were only present in IgAV-M and IgAV-C. We also found four genera, including *Enterococcus*, *Weissella*, *Megamonas* and *Lactobacillus*, were significantly enriched in both IgAV-C and IgAV-M groups, compared with H-C and H-M (Supplementary Data 3). The relative abundance of *Megamonas* (*r* = 0.6759, *P* < 0.001, Figure 3B) and *Lactobacillus* (*r* = 0.2138, *P* < 0.05, Figure 3B) in IgAV children was positively correlated with that in mothers using Spearman Correlation Coefficient.

Through the Neighbor-Joining method, the phylogenetic analyses respectively based on all ASVs in two genera mentioned above, also revealed that ASVs of the same genus, in IgAV-C and IgAV-M groups, were mostly shared and extremely close to each other (Figure 3C). Overall, these findings suggest that the microbiota of mothers, whose child suffered from IgAV, are similar to their kids, suggesting the substantial impact of mothers on shaping their children's microbiota.

### 3.4 Microbial biosynthesis of vitamins and essential amino acids is upregulated, whereas monosaccharide degradation pathways are downregulated in children with IgAV

**Figure 4:**
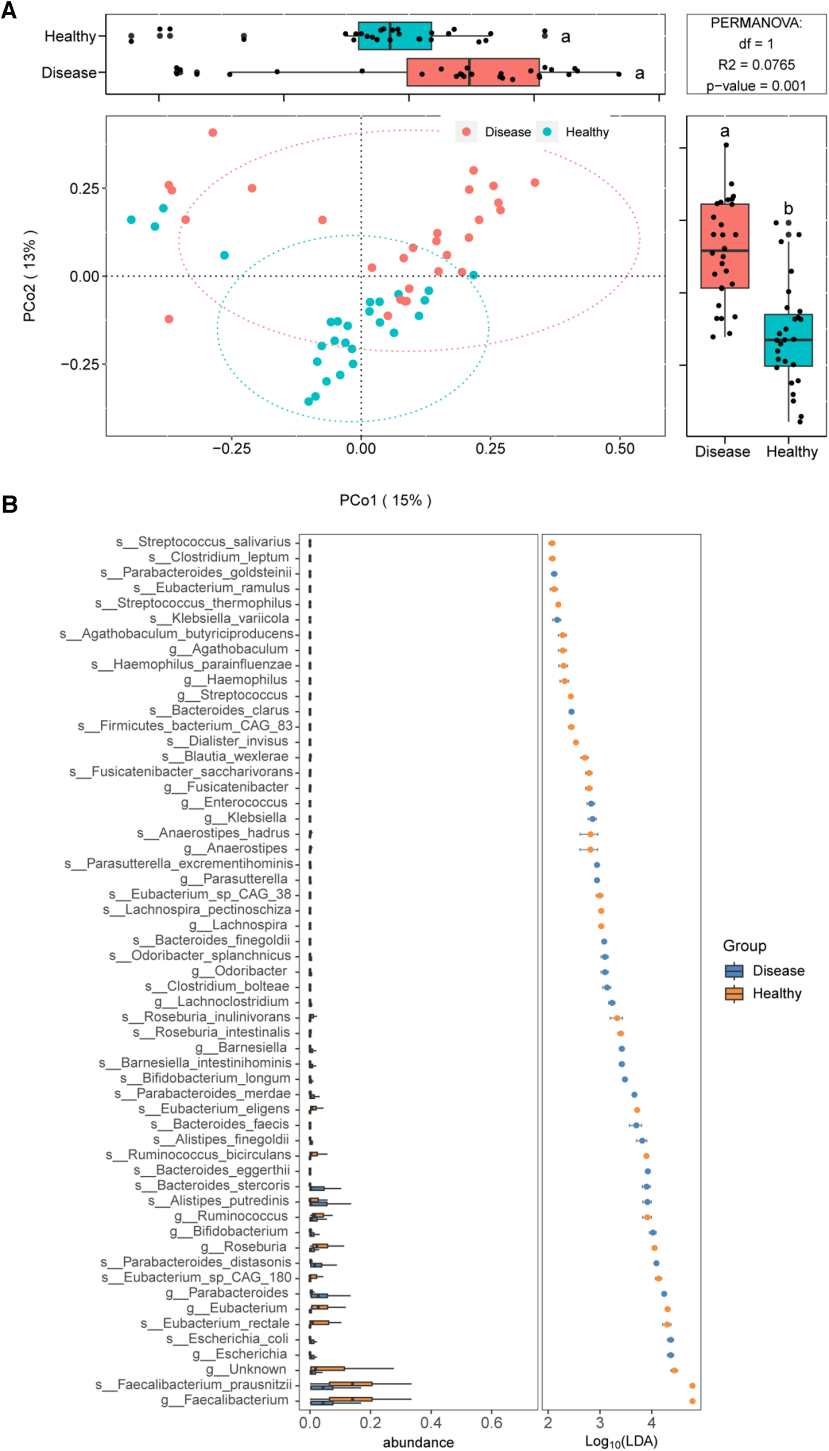
Distinct gut microbiomes were observed in IgAV children (IgAV-C) compared to healthy controls (H-C) with shotgun metagenomic sequencing. (**A**) PCoA and boxplot are shown along the first two principal coordinates of Bray-Curtis distances for disease group (include 28 IgAV children) and healthy group (include 27 healthy children). (**B**) Significantly different abundant taxa with LDA score (log10) >2.0 and *P* < 0.05 at the species or genera level, between disease and healthy groups.

We employed metagenomic sequencing to further characterize the gut bacterial community structure in children with IgAV. The beta diversity analysis also suggested obvious differences in bacterial communities among IgAV children and healthy control (Figure 4A).

We further identified gut microbial taxa associated with IgAV. In discovery cohort (*n* = 55), a total of 10 phyla, 118 genera and 275 species were identified (Supplementary Data 4). At the phylum level, *Bacteroidetes* and *Proteobacteria* were increased in IgAV patients, whereas *Firmicutes* was decreased (LDA>2; Supplementary Data 5). The abundance of 19 genera and 37 species exhibited significant differences between IgAV children and controls (Figure 4B).

**Figure 5:**
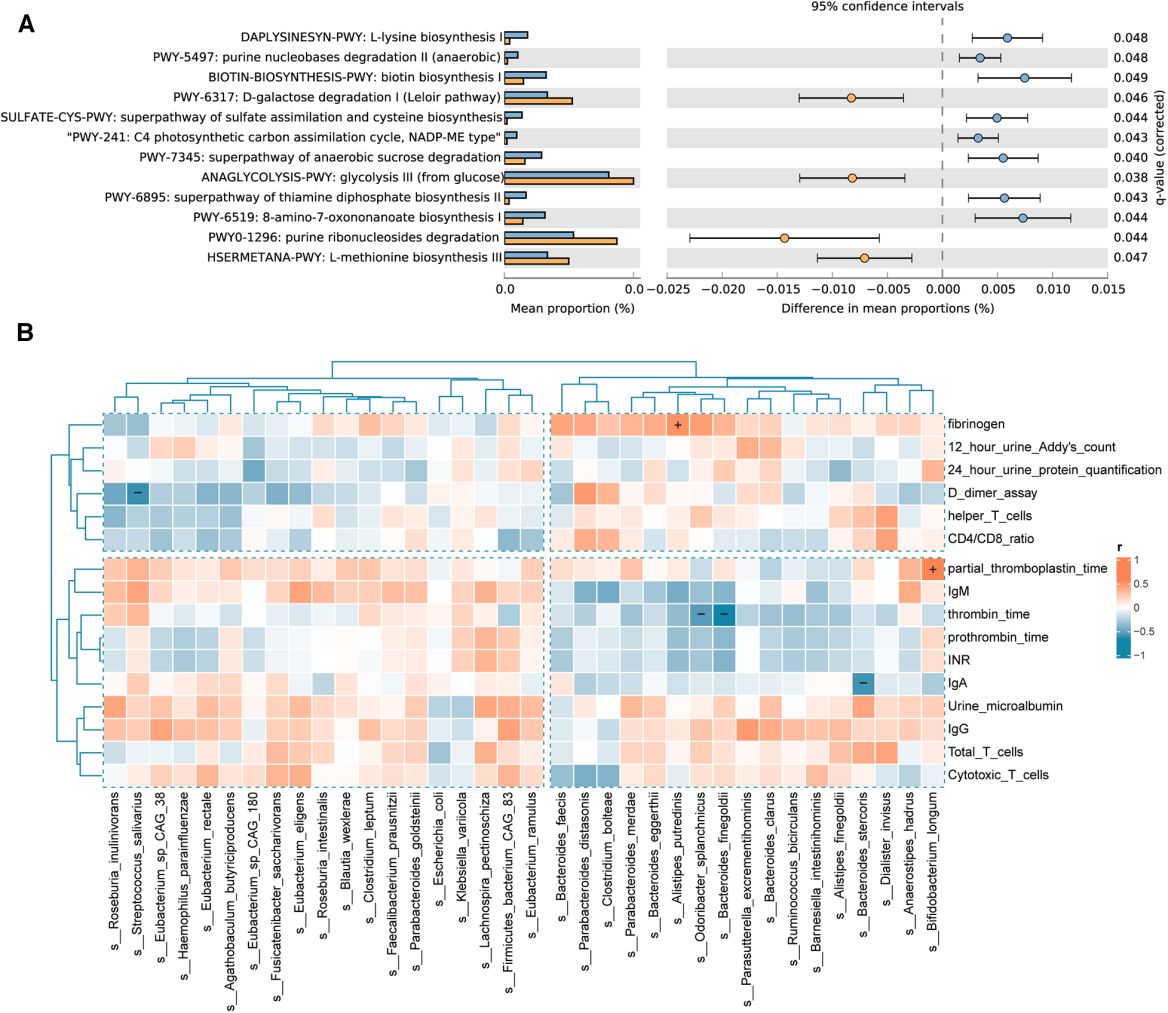
Differential functional pathways in the gut microbiota and correlation of gut microbiota with clinical indicators. (**A**) The relative abundance of differential functional pathways in the gut microbiota. The barplot with 95% confidence intervals denote the significantly different microbial pathways between IgAV children and controls. Blue, disease group; yellow, healthy group. (**B**) Heatmap of Spearman correlation analysis between the gut microbiota and clinical indicators. The results suggested that clinical indicators are associated with differential bacterial abundance. Red and blue indicate positive and negative correlations, respectively. (+/-, *P* < 0.05).

The results of the pathway enrichment analysis demonstrated statistical differences (*P* < 0.05) in 12 metabolic pathways between the IgAV and control groups (Figure 5A). Microbial pathways related to vitamin biosynthesis and essential amino acid biosynthesis are upregulated, whereas monosaccharide degradation pathways are downregulated in Children with IgAV. The upregulated microbial pathways related to the biosynthesis of vitamins in IgAV include (i) B1/thiamine [superpathway of thiamine diphosphate biosynthesis II (PWY-6895)], and (ii) B7/biotin [biotin biosynthesis I (BIOTIN-BIOSYNTHESIS-PWY)]. Children with IgAV exhibited an upregulated microbial biosynthesis of essential amino acid such as L-lysine [L-lysine biosynthesis I (DAPLYSINESYN-PWY)] and L-methionine [L-methionine biosynthesis III (HSERMETANA-PWY)]. Two glycatabolism pathways are downregulated in children with IgAV: (i) glycolysis III (from glucose) (ANAGLYCOLYSIS-PWY) and (ii) D-galactose degradation I (Leloir pathway) (PWY-6317). Several microbial precursor metabolites and energy pathways were also altered between healthy children and children with IgAV (Figure 5A).

### 3.5 Bacterial species differed between IgAV and healthy children are associated with clinical factors

To examine the potential links between microbial community composition and clinical variables, the following three types of clinical variables were chosen for correlation analysis, indicators of IgAV nephritis, immunity, and blood coagulation. Additionally, the Spearman correlation heatmap illustrated the relationships between clinical variables and bacterial biomarkers. As depicted in Figure 5B, indicators of blood coagulation, including D-dimer assay, thrombin time, partial thromboplastin time (PPT) and fibrinogen, are associated with the intestinal community composition in children with IgAV. The abundance of *Bifidobacterium longum* showed a positive correlation with PPT. Similarly, the abundance of *Alistipes putredinis* was positively correlated with fibrinogen levels. Conversely, the abundance of *Odoribacter splanchnicus* and *Bacteroides finegoldii* exhibited a negative correlation with thrombin time. Notably, we also found a negative correlation between the abundance of *Bacteroides stercoris* and the important immune factor IgA.

## 4 Discussion

New studies have substantiated that the manifestation of IgAV is affected by both genetic and environmental factors (22). However, the investigation of environmental factors potentially contributing to the development of IgAV and the underlying mechanisms is still in its early stages. In our previous study, we identified a dysbiosis in the gut microbiota of children who were diagnosed with IgAV (17, 23). Given the high prevalence and severe manifestations of IgAV, it is crucial to comprehend the interactions between the host and the microbiome, as well as the alterations in the microbiome that contribute to dysbiosis in IgAV. Metagenomic shotgun sequencing provided a means to analyze and characterize the composition and functional profile of the gut microbiome. And considering of maternal factors can help to better understand the shaping of the microbiome in children with diseases.

Our study revealed significant dysbiosis in the gut microbiota of children with IgA vasculitis and their mothers, characterized by distinct alterations in microbial composition compared to healthy children and their mothers. Furthermore, there were strong correlations between the microbiological profiles of mothers and their children, although the IgAV children also showed distinct bacterial markers.

First of all, the findings obtained from 16S rRNA gene sequencing provided evidence that four genera, namely *Lachnospira*, *Ruminococcus*, *Roseburia* and *Streptococcus*, exhibited a reduced relative abundance in IgAV patients. These results align with the outcomes acquired from our metagenomics analysis. Similar findings were observed for the *Enterococcus* genus (and its parent family *Enterococcaceae*) in both 16S and metagenomic analysis, but in the opposite direction. Thus, there is considerable similarity in the gut microbiota markers of IgAV between the two analyses employing distinct methodologies.

We also discovered a distinct set of bacterial biomarkers that exhibited significant variations between the two groups. For instance, *Enterococcus*, a bacterium known to potentially contribute to intestinal infections in IgAV patients with microbiota dysbiosis, was among the identified biomarkers (17). Likewise, the bacterial genera *Dialister* and *Roseburia* were notably reduced in abundance in the IgAV children group. *Dialister* abundance has also been reported to be inversely associated with eczema. *Dialister* and *Roseburia*, similar to certain *Clostridia* species that possess the capacity to produce SCFAs, are believed to have anti-inflammatory properties. A reduction in *Roseburia* also had been observed in patients diagnosed with ulcerative colitis. It can product butyrat (24), which plays a crucial role in preserving the health of the colonic mucosa and promoting anti-inflammatory effects (25, 26). Additionally, we observed a lower abundance of *Ruminococcus* in IgAV children. A recent study also found that the abundance of *Ruminococcus* increased in the convalescent stage of IgAV patients (27). Therefore, the decrease in the abundance of *Ruminococcus* is closely related to the changes in the immune response of the children. A reduction in the abundance of the *Ruminococcus* genus may result in decreased production of butyrate and propionate, as well as diminished anti-inflammatory activity of short-chain fatty acids (SCFAs), ultimately leading to abnormal Th2 immune responses.

As anticipated, there were dramatical differences in the gut microbiome between the IgAV-M and H-M groups. Significantly, we identified a remarkable correlation between the microbiomes of mother-child pairs. We found IgAV-C and IgAV-M share common biomarkers, such as *Enterococcus*, *Weissella*, *Megamonas* and *Lactobacillus*, whereas 13 genera, which include *Lachnospira*, *Fusicatenibacter*, *Monoglobus* and *Clostridia_UCG-014*, were decreased in both IgAV-C and IgAV-M group. *Enterococcus* is a prominent commensal bacterium found in the human gastrointestinal tract, which was found to be associated with acquired immunodeficiency syndrome (28), such as IgAV (17) and Crohn's disease (29). *Enterococcus* is an important opportunistic pathogen that typically resides in the human intestinal tract without causing any symptoms in healthy individuals. However, when there is a disruption in the balance of the microbiota (known as dysbiosis), *Enterococcus* can become pathogenic and cause infections. Previous studies have shown that the existence of *Lachnospira* can promote the equilibrium of beneficial bacterial communities within the intestinal tract, thus maintaining a stable gut microbiota (30). It actively participates in the degradation and metabolism of polysaccharides during the process of digestion, leading to the production of beneficial short-chain fatty acids like propionic acid and butyric acid (10). These short-chain fatty acids not only supply energy to intestinal mucosal cells but also possess anti-inflammatory and immune regulatory properties. The consistent changes in these harmful or beneficial bacteria between IgAV-C and IgAV-M may not be coincidental. Gut microbiome can vertical transmission from the mother to the gut of offspring, which could offer a partial explanation for the similarities seen in the gut microbiome compositions of mother-child pairs (31). According to certain experts, there is a growing belief that the colonization and development of the newborn microbiota might initiate during the *in utero* phase (32). This suggests that the appropriate modifications of gut microbiota in children could be influenced by earlier changes in the maternal microbiota, which may occur during pregnancy or even before pregnancy (33). So, it is meaningful to take maternal factors into account when fully understanding the characteristics of IgAV-C microbiota.

Additionally, our research has found that, microbial biosynthesis of vitamins and essential amino acids is upregulated, whereas monosaccharide degradation pathways are downregulated in IgAV-C. Therefore, IgAV patients may experience deficiencies in vitamins and essential amino acids, leading to changes in microbial metabolic pathways. Such as systemic small vessel inflammation leads to increased vitamin consumption and demand, while gastrointestinal ulcers lead to reduced vitamin absorption (34, 35). Our research has also found that changes in the gut microbiota may affect coagulation function. Significantly enriched *Odoribacter splanchnicus* and *Bacteroides finegoldii* in IgAV patients showed a negative correlation with thrombin time, while *Alistipes putredinis* exhibited a positive correlation with fibrinogen levels. These correlation results collectively suggested that some of the significantly enriched microorganisms in IgAV patients may be associated with pathological reactions related to coagulation and fibrinolysis hyperfunction. Currently, there is still limited direct evidence regarding the direct relationship between the gut microbiota and host coagulation function. However, some evidence has proposed metabolites produced by certain bacteria can influence the synthesis and activity of clotting factors, leading to abnormal coagulation function (36, 37). A possible mechanism by which gut microbiome dysbiosis may affect coagulation is through low-grade inflammation driven by translocation of LPS from gram-negative bacteria in the gut to the systemic circulation (38). Furthermore, intravenously administered LPS has been demonstrated to increase procoagulant factors, in healthy volunteers (39). Moreover, Potential intestinal pathogens can also induce intestinal mucosal inflammation and release pro-inflammatory cytokines. Inflammatory reactions have the potential to enhance coagulation activity, which in turn elevates the likelihood of thrombosis (40). It is important to note that the above evidence only provides potential associations between the gut microbiota and host coagulation function, and further research is needed to validate this relationship.

To sum up, the research content in this paper investigated the characteristics of the gut microbiota in IgAV children and their interrelationships with the host, while exploring the vertical inheritance of the microbiota in IgAV-M. We found the IgAV children and their mothers exhibit community structure changes of gut microbiome, where *Enterococcus*, *Weissella*, *Megamonas* and *Lactobacillus* become the predominant bacteria. Alterations in both the quantity and structure of the bacterial community may play a role in the development of IgA vasculitis and could also serve as a marker of disease progression. However, due to the cross-sectional design of the study, the underlying mechanisms and longitudinal aspects of the observed correlations could not be fully understood. Further extensive cohort studies are necessary to establish the chronological order and assess alterations in the gut microbiome of children with IgAV and their mothers.

## 5 Conclusions

Children with IgA vasculitis have unique bacterial biomarkers and may affect coagulation function, and their gut microbiome was closely associated with that of their mothers. The observed association in gut microbiota between IgA vasculitis children and their mothers suggested a potential intergenerational influence of the maternal microbiota on the development or progression of IgA vasculitis in children.

## Data Availability

All raw sequencing data have been deposited into the National Omics Data Encyclopedia (NODE; https://www.biosino.org/node/index) with the accession number OEP004691.
